# Spontaneous Recovery of Paraplegia in a Polytrauma Patient following Spinal Cord Ischemia due to Type B Traumatic Aortic Dissection

**DOI:** 10.1155/2023/8918724

**Published:** 2023-08-11

**Authors:** Pedro Ramos Jurado, Fernando Hernández Aragón, Víctor Aaron Miranda González, Jesús Antonio Loya Silva, Edgar Azael Pérez Gutiérrez, Nadia Karina Portillo Ortiz, Adriana Cristina Quintana Vázquez, Luisa Fernanda Trujillo Venzor, Eduardo Enrique Gámez Aponte, Arturo Aguirre Madrid, Edmundo Berumen Nafarrate

**Affiliations:** ^1^Department of Orthopedic Surgery, Christus Muguerza del Parque Hospital, De la Llave Street No. 1419, Office 9, Col. Centro, Chihuahua 31000, Mexico; ^2^Department of Angiology and Vascular Surgery Hospital Ángeles Chihuahua (CIMA Hospital), C. Haciendas del Valle 7120-Interior 21, Haciendas del Valle III, Chihuahua, 31217 Chih, Mexico; ^3^Faculty of Medicine and Biomedical Sciences, University Autonomous of Chihuahua (UACH), Chihuahua, Mexico; ^4^Universidad de Monterrey (UDEM), Ignacio Morones Prieto Avenue 4500 W, San Pedro Garza Garcia, Nuevo Leon 66238, Mexico

## Abstract

Aortic dissection is a life-threatening acute condition characterized by the separation of the aortic wall's layers. It is caused by a tear in the internal vascular wall (intimal layer and middle layer), which results in bleeding between the layers and causes abrupt and excruciating pain. The appropriate consideration must be given to the condition's dynamic nature, and variations in clinical presentation, without neglecting the urgency for intervention. In this case study, a 65-year-old male engaged in a car accident is admitted to urgent care with a traumatic aortic dissection diagnosis that included the aortic arch, a segmental exposed fracture of 1/3 distal of the right femur AO 32C3k, and an intertrochanteric fracture AO 31A1.3. The patient developed transient paraplegia as the initial manifestation of acute aortic dissection, which represents a high mortality and morbidity entity without adequate and prompt treatment, and prompt diagnosis and management were critical. A patient with severe thoracic and abdominal trauma caused by high-energy injury should be properly evaluated for the possibility of traumatic aortic dissection. The endovascular aortic repair was performed, resulting in a positive clinical evolution due to the important participation of the multidisciplinary trauma team involved in patient management and prompted decision-making.

## 1. Introduction

Type B aortic dissection affects the intimal layer of the arteries, which allows the extravasation of blood and dissection to the middle layer. Its incidence is up to 6 cases per 100,000 inhabitants-year, with an increase, especially in patients over 65 years of age [1].

The most catastrophic event related to the pathology is the rupture and occlusion of arterial branches, with the consequent loss of volume and hypovolemic shock. Mortality rates are very high, and factors that predispose to death are the presence of previous pathologies [2, 3].

Another uncommon but possible complication is the presence of paraplegia or paresthesia associated with the involvement of spinal cord circulation; this is present in up to 7% of patients. Alteration of the arterial flow of the spinal cord is caused secondary to separation or occlusion of intercostal arteries at the aortic lumen, thrombosis, and hypoperfusion associated with the flow of the blood to the false lumen [4–6]. A less frequent complication is lumbosacral plexopathy, associated with altered blood flow to the nervorum artery or by occlusion of the iliac branches [7, 8].

We present a rare case of aortic dissection type IIIb classification DeBakey, Stanford type b associated with fracture of the right hip and femur, with the recovery of paraplegia secondary to effective intervention through the placement of endovascular prostheses.

## 2. Clinical Case

**Figure 1:**
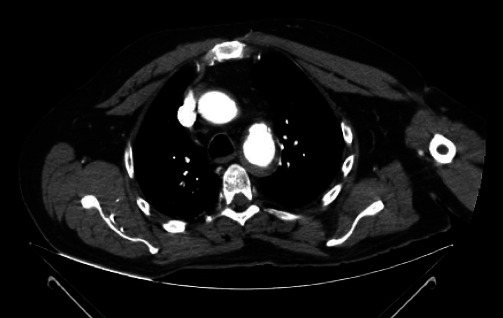
Axial computed tomography scan of the chest performed on a 65-year-old male patient after a frontal collision accident. Aortic dissection is observed at the level of the arch and proximal third of the descending aorta.

A 65-year-old male suffers a frontal collision accident against a cargo trailer, he confirms wearing a seat belt, denies loss of consciousness in the event, and is taken to the emergency unit. The patient arrives at the emergency department with stable vital signs, dyspnea, precordial, and upper abdominal pain. Hypoesthesia at the level T10 of pelvic limbs with predominance on the left side, ASIA force score 2/5 and sensitivity 1/2 at T10 level, and exposed fracture at the level of the distal femur is documented. After initial stabilization, computed tomography is performed due to suspicion of a complete spinal cord section. Results show evident injury at the level of the aortic arch and descending portion, without data of spinal compromise (Figure 1).

**Figure 2:**
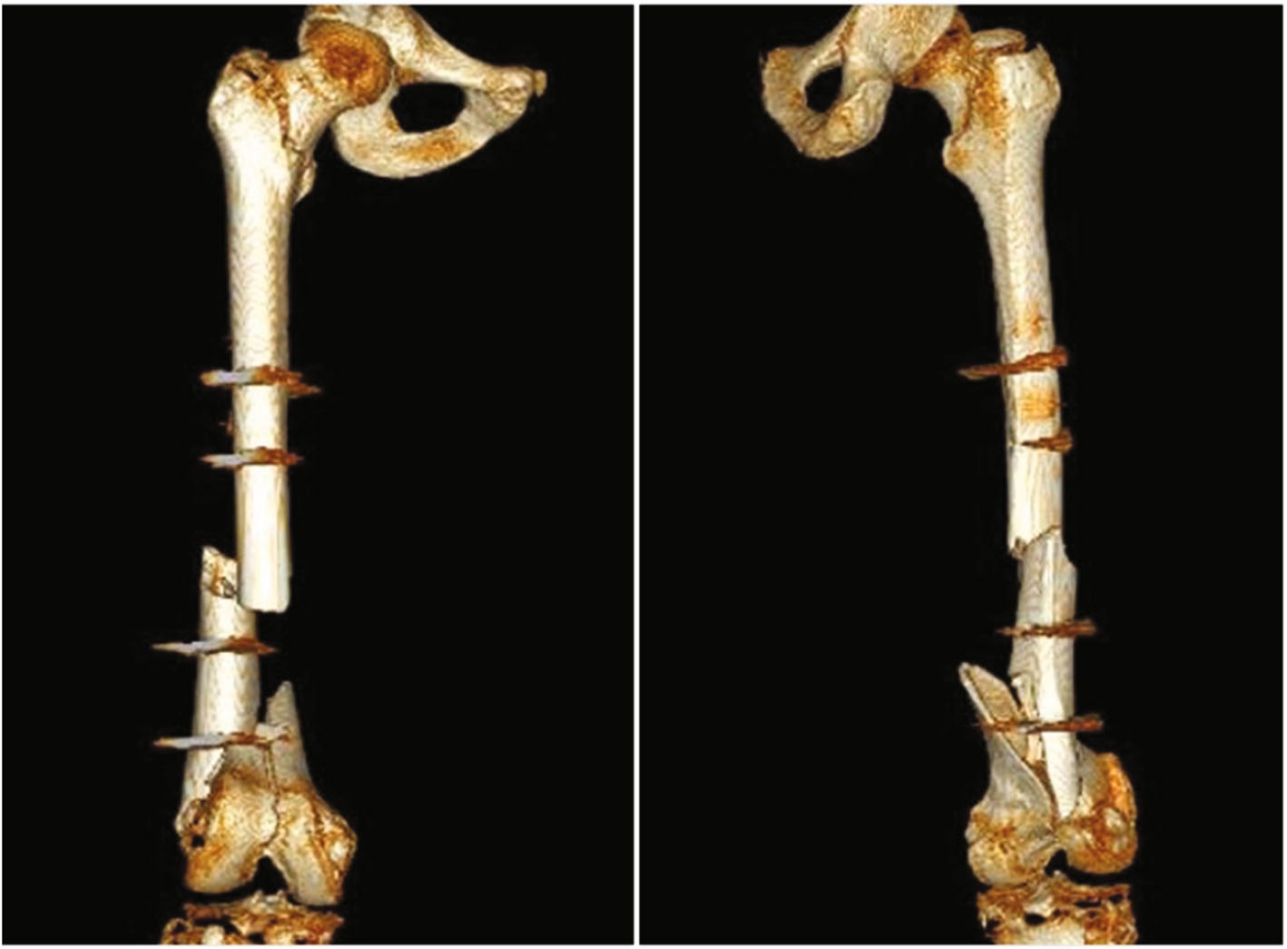
3D reconstruction of a segmental fracture of the distal 1/3 of the right femur AO 32C3k (treated with external fixators) and intertrochanteric fracture AO 31A1.3.

Surgical cleaning of exposed fractures of the distal femur and placement of external fixators is performed due to the risk of clamping of the popliteal artery. It is worth mentioning that this procedure is performed without anesthesia due to the paraplegia presented by the patient (Figure 2). After stabilization, he is transferred to a hospital with a hemodynamic area available.

Upon arrival, the patient refers to pain in the precordial region and dyspnea. Vital signs were heart rate (HR) of 66 bpm, respiratory rate of 22 rpm, temperature 36.6°C, average blood pressure (BP) of 66 mmHg, and BP of 90/55 mmHg.

During the physical examination, the patient was alert, orientated, and cooperative, right eye with papilledema and upper hematoma, consensual light reflex was present, and a full examination of cranial pairs III, IV, and IV was performed without abnormality. Symmetrical chest, respiratory movements limited by pain, rhythmic heart sounds, normal tone, intensity, and frequency. Soft abdomen, referring pain at palpation in hypogastrium, without data of peritoneal irritation, negative Blumberg sign. Lower limbs presented posterior tibial and pedal pulses of good intensity; left pelvic limb with loss of strength, ASIA score strength 2/5, sensitivity 1/2, and presence of external fixators in the right femur.

The laboratory values obtained were as follows: hemoglobin 8.0 g/dL, hematocrit 24.4%, leukocytes 15.63 K/*μ*L, neutrophils 14.20 K/*μ*L, platelets 94 K/*μ*L, glucose 171 mg/dL, urea 61.0 mg/dL, blood urea nitrogen 13.36 mg/dL, creatinine 1.55 mg/dL, arterial gasometry showed pH 7.36, pCO_2_ 43.0 mm/Hg, pO_2_ 77.0 mm/Hg, lactate 2.2 mmol/dL, sodium 137.0 mmol/L, potassium 3.97 mmol/L, and ionic calcium 1.1 mmol/L.

Imaging studies confirm aortic dissection at the level of the arch and proximal third of the descending aorta, segmental fracture of 1/3 distal of the right femur AO 32C3k (treated with external fixators), and intertrochanteric fracture AO 31A1.3.

**Figure 3:**
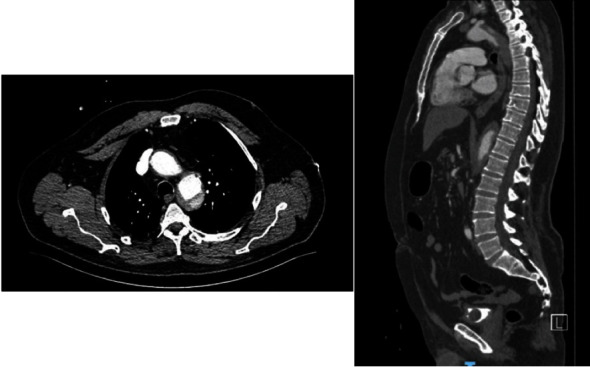
Axial and sagittal section of the thorax and abdomen with intravenous contrast, with angiotomography protocol where dissection is observed at the level of the aortic arch, distal to the emergence of the supra-aortic stems (DeBakey III) with extravasation of the contrast medium into the false lumen, and extension until the emergence of the celiac trunk.

Angiotomography is requested by vascular surgery, showing contrast exit at the level of the aortic arch through the false lumen. The aortic dissection showed extension from the aortic arch after the emergence of the left subclavian vessel to the emergence of renal arteries. The dissection is classified as type IIIb DeBakey, Stanford type B (Figure 3). Global cardiomegaly and bilateral pleural effusion with associated subsegmental atelectasis were also reported.

**Figure 4:**
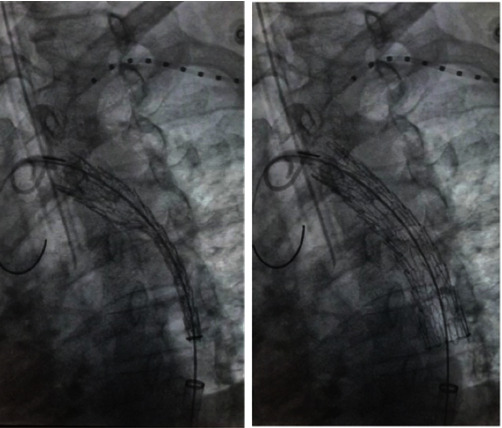
Fluoroscopic image of endovascular prosthesis placement.

Urgent intervention is decided. Endovascular prostheses placement in the hemodynamics area is decided (Figure 4). Without complications during the event, the stable patient passes to the intensive care unit, with oxygen support by nasal tips at 5 L/minute, saturating 99%, and thromboprophylaxis is initiated with enoxaparin. On the immediate post-surgical day, the patient is hemodynamically stable, without amines, conscious and oriented, and two globular units of 22% are transfused.

Venous Doppler ultrasound of the lower extremities is performed, and soft tissue edema predominance in the thigh's proximal third is observed. The report shows high resistance indices in all the evaluated segments of the arterial system and the venous system, without evidence of superficial or deep thrombosis. Patient care continues without sedation, supplementary O_2_ support with nasal tips at 2 L/minutes saturating 99%, dynamically stable blood with the medial arterial pressure 80–85 mmHg, BP 132/65 mmHg, HR 55 bpm, without aminergic support, nitroglycerin infusion 4 cc/hour, and base solution 80 cc/hour.

**Figure 5:**
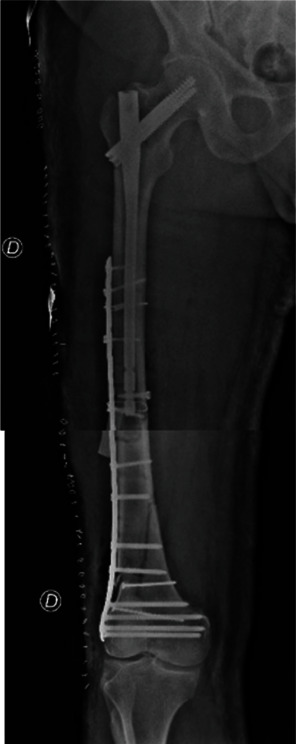
Post-surgical X-ray of the right femur after osteosynthesis with proximal femoral nail and distal femur anatomical plate.

Three days after admission, the osteosynthesis of the right femur is performed with proximal femoral nail and anatomical plaque of the distal femur (Figure 5), without incidents or accidents during surgery, after the surgical event a globular package is transfused. Treatment with quetiapine is added, exercises are started with an incentive spirometer, and two platelet apheresis is transfused. It was decided to administer an alpha-blocker, Prazosin, at 1 mg every 6 hours. The vascular surgery team places a pneumatic mattress to prevent ulceration.

On his fifth day of in-hospital stay, active mobilization of the left pelvic limb begins, an improvement in force is observed with a 4/5 in ASIA SCORE (on admission 2/5) sensitivity 2/2, capillary blood filling in less than two seconds in both pelvic limbs, and surgical wound in the right femur without data of dehiscence or active bleeding.

Mesenteric angiography is repeated, reporting the heart of normal morphology and dimensions. The aortic arch was identified with a vascular endoprosthesis that extended towards the descending aorta, with no changes in its position when compared with previous studies. After administration of the contrast medium, a hypodense intimal flap was identified without evidencing contrast medium output, which extended from the bifurcation of the left subclavian artery to the bifurcation of the renal arteries.

**Figure 6:**
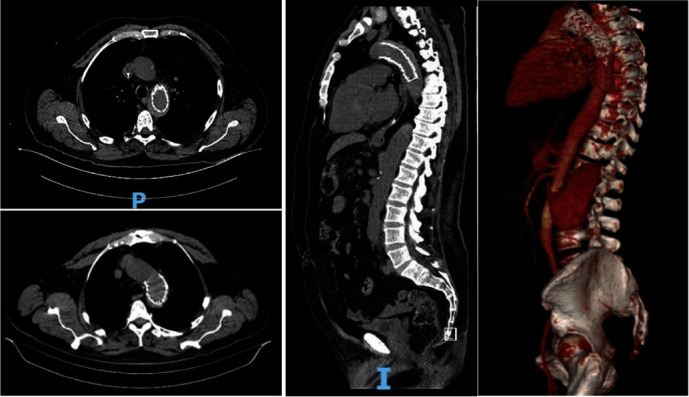
Post-surgical angiomesenteric tomography, an aortic endoprosthesis is observed in the aortic arch and descending portion. No data on intramural hematomas or extravasation of contrast at the time of the study.

Aortic artery with a diameter of 30 mm in its ascending portion and descending aorta with a diameter of 34 mm without evidence of filling defects throughout its length (Figure 6).

Prazosin is suspended, the patient is capable to move out of bed, and laboratories are requested as follows: hemoglobin 10 mg/dL, hematocrit 30.3%, leukocytes 8.76 K/*μ*L, platelets 53 K/*μ*L, and creatinine 1.0 mg/dL. Platelet apheresis is transfused, chest and abdominal tomography are performed, general urine test with negative ketones, and no bacteria are observed.

Hemoculture of the central venous catheter showed a positive result for *Staphylococcus hominis* ssp. hominins resistant to clindamycin and erythromycin, the catheter is removed, and antibiotic treatment is initiated with trimethoprim–sulphamethoxazole. Negative right-arm hemoculture after five days, negative urine culture at 72 hours, and negative catheter tip cultivation at 72 hours were noted. The patient continues in good general condition, tolerates diet, does not refer pain, without secretion in the wound, continues afebrile, has stable vital signs, and cardiopulmonary examination without abnormalities, antibiotic management is continued at home after the patient is discharged by the angiology service, and follow-up by external consultation.

Through several surgical procedures, post-surgery recovery control was maintained during the patient's hospital stay through daily surveillance of the clinical condition, pain control, laboratory analysis, imaging scans, and rehabilitation treatment, as well as early diagnosis of an infection associated with central venous access, with opportune antibiotic treatment, as the only incident. Following hospital discharge, the final post-surgical follow-up assessment was performed at 60 days, during which an excellent clinical evolution could be observed, with total strength recovery evaluated with an ASIA score of 5/5, sensitivity 2/2, the patient remains at all times with restricted support in the affected limb, in continuous mobilization with a wheelchair to carry out daily activities, this to favor the consolidation of the fracture.

## 3. Discussion

Paraplegia in a polytrauma patient could be the main symptom of wide vascular syndromes affecting the spinal cord, lumbosacral roots, and lumbosacral plexus by ischemia of the peripheral nerves. Onset and clinical manifestation depend on the anatomical site and grade of vascular injury [6].

The vascular plexus is incorporated into the psoas in its back and can give a certain supply of blood flow. However, the main vascular supply of the plexus originates from the internal iliac artery that originates the iliolumbar artery, which provides a lumbar branch. The lumbar plexus rarely suffers alterations in circulation because it has multiple collateral arteries from the psoas muscle that complement the vascular [9–11]. The theory that supports the presence of this collateral circulation is based on the small incidence of lumbar plexopathy after aortoiliac reconstruction [12–14]. Several cases have been reported associated with acute and chronic thrombotic events, also due to prolonged hypotension [15–18].

The importance of being able to distinguish the type of damage that the spinal cord has suffered is one of the main determinants in the patient's prognosis, a multidisciplinary assessment, and emergency treatment are part of the comprehensive assessment of patients. Isolated ischemic lesion of the lumbosacral plexus has a better prognosis than spinal cord infarction, so recognizing clinical data in a patient with aortic dissection and paraplegia makes the need for endovascular intervention to prevent devastating complications.

In the isolated ischemic of the lumbosacral plexus due to the more distal involvement, the motor neurons of the marrow are preserved and there is some possibility of recovery of their axons once the circulation has been restored [19]. However, many current studies discuss the low possibility of this recovery. We infer that the paraplegia the patient will present was related to hypoperfusion due to the blood outflow to the false lumen and the extension of aortic dissection from his admission to the medical unit, which he was received and as progress on his arrival at our hospital.

On the one hand, the presence of collateral circulation prevented progression to spinal cord infarction and contributed as a good prognostic factor for the patient's recovery. On the other hand, we do not rule out as a second cause a SCWIORA related to the traumatic event that the patient suffered. However, we could not rule out multiple ischemic neuropathies (distant from the plexus) of the lower extremities that could imitate a bilateral involvement of the plexus.

## 4. Conclusion

The complete evaluation of the polytraumatized patient is important, as well as the clinical suspicion of injuries associated with fractures that translate a large amount of energy into car accidents. The complete assessment through the ASIA SCORE should be routine in the emergency services for patients with neurological changes. It is of the utmost importance to know the anatomy of the spinal cord to determine our patients' possible changes and clinical manifestations. The damage control protocol is another concept to be taken into account in decision-making for the definitive management of these patients. The presence of a multidisciplinary team, imaging, and laboratory studies are of the utmost importance, they help to achieve more accurate diagnoses and improve the prognosis and quality of life of our patients.

## Data Availability

The authors confirm that the data supporting the information of this clinical case study were derived from the following resources available in the public domain.
